# Genome-Wide Analysis of SREBP1 Activity around the Clock Reveals Its Combined Dependency on Nutrient and Circadian Signals

**DOI:** 10.1371/journal.pgen.1004155

**Published:** 2014-03-06

**Authors:** Federica Gilardi, Eugenia Migliavacca, Aurélien Naldi, Michaël Baruchet, Donatella Canella, Gwendal Le Martelot, Nicolas Guex, Béatrice Desvergne, the CycliX Consortium, co-PIs, Mauro Delorenzi, Bart Deplancke, Béatrice Desvergne, Nicolas Guex, Winship Herr, Felix Naef, Jacques Rougemont, Ueli Schibler, Management committee, Bart Deplancke, Nicolas Guex, Winship Herr, Bioinformatics coordination, Nicolas Guex, Animal breeding, conditioning, collections of biological material, Teemu Andersin, Pascal Cousin, Federica Gilardi, Pascal Gos, Gwendal Le Martelot, Fabienne Lammers, Chromatin immunoprecipitations, libraries, gene expression arrays, Donatella Canella, Federica Gilardi, Sunil Raghav, Computing infrastructure installation and servers maintenance, Roberto Fabbretti, Arnaud Fortier, Li Long, Volker Vlegel, Ioannis Xenarios, Tag mapping, quantification, and normalizations, Eugenia Migliavacca, Viviane Praz, Nicolas Guex, Felix Naef, Jacques Rougemont, Data management and viewing tools, Fabrice David, Yohan Jarosz, Dmitry Kuznetsov, Robin Liechti, Olivier Martin, Julien Delafontaine, Lucas Sinclair, Bioinformatics: RNA polymerase II and histone marks, Julia Cajan, Irina Krier, Marion Leleu, Eugenia Migliavacca, Nacho Molina, Aurélien Naldi, Guillaume Rey, Laura Symul, Nicolas Guex, Felix Naef, Jacques Rougemont, Bioinformatics: RNA polymerase III and histone marks, David Bernasconi, Mauro Delorenzi, Molecular and cellular biology, biochemistry, Teemu Andersin, Donatella Canella, Federica Gilardi, Gwendal Le Martelot, Fabienne Lammers, Michaël Baruchet, Sunil Raghav

**Affiliations:** 2) Swiss Institute of Bioinformatics, University of Lausanne; 7) Bioinformatics Core Facility, Swiss Institute of Bioinformatics; 8) Department of Oncology and Ludwig Center for Cancer Research, Faculty of Biology and Medicine, University of Lausanne; 3) The Institute of Bioengineering (IBI), School of Life Sciences, Ecole Polytechnique Fédérale de Lausanne (EPFL); 1) Center for Integrative Genomics, Faculty of Biology and Medicine, University of Lausanne; 4) Vital IT, Swiss Institute of Bioinformatics; 1) Center for Integrative Genomics, Faculty of Biology and Medicine, University of Lausanne; 3) The Institute of Bioengineering (IBI), School of Life Sciences, Ecole Polytechnique Fédérale de Lausanne (EPFL); 5) Bioinformatics and Biostatistics Core Facility, School of Life Sciences, Ecole Polytechnique Fédérale de Lausanne (EPFL); 6) Department of Molecular Biology, Faculty of Sciences, University of Geneva; 3) The Institute of Bioengineering (IBI), School of Life Sciences, Ecole Polytechnique Fédérale de Lausanne (EPFL); 4) Vital IT, Swiss Institute of Bioinformatics; 1) Center for Integrative Genomics, Faculty of Biology and Medicine, University of Lausanne; 4) Vital IT, Swiss Institute of Bioinformatics; 6) Department of Molecular Biology, Faculty of Sciences, University of Geneva; 1) Center for Integrative Genomics, Faculty of Biology and Medicine, University of Lausanne; 1) Center for Integrative Genomics, Faculty of Biology and Medicine, University of Lausanne; 6) Department of Molecular Biology, Faculty of Sciences, University of Geneva; 6) Department of Molecular Biology, Faculty of Sciences, University of Geneva; 1) Center for Integrative Genomics, Faculty of Biology and Medicine, University of Lausanne; 1) Center for Integrative Genomics, Faculty of Biology and Medicine, University of Lausanne; 1) Center for Integrative Genomics, Faculty of Biology and Medicine, University of Lausanne; 3) The Institute of Bioengineering (IBI), School of Life Sciences, Ecole Polytechnique Fédérale de Lausanne (EPFL); 4) Vital IT, Swiss Institute of Bioinformatics; 4) Vital IT, Swiss Institute of Bioinformatics; 4) Vital IT, Swiss Institute of Bioinformatics; 4) Vital IT, Swiss Institute of Bioinformatics; 1) Center for Integrative Genomics, Faculty of Biology and Medicine, University of Lausanne; 2) Swiss Institute of Bioinformatics, University of Lausanne; 4) Vital IT, Swiss Institute of Bioinformatics; 4) Vital IT, Swiss Institute of Bioinformatics; 1) Center for Integrative Genomics, Faculty of Biology and Medicine, University of Lausanne; 4) Vital IT, Swiss Institute of Bioinformatics; 3) The Institute of Bioengineering (IBI), School of Life Sciences, Ecole Polytechnique Fédérale de Lausanne (EPFL); 5) Bioinformatics and Biostatistics Core Facility, School of Life Sciences, Ecole Polytechnique Fédérale de Lausanne (EPFL); 2) Swiss Institute of Bioinformatics, University of Lausanne; 5) Bioinformatics and Biostatistics Core Facility, School of Life Sciences, Ecole Polytechnique Fédérale de Lausanne (EPFL); 2) Swiss Institute of Bioinformatics, University of Lausanne; 5) Bioinformatics and Biostatistics Core Facility, School of Life Sciences, Ecole Polytechnique Fédérale de Lausanne (EPFL); 4) Vital IT, Swiss Institute of Bioinformatics; 4) Vital IT, Swiss Institute of Bioinformatics; 4) Vital IT, Swiss Institute of Bioinformatics; 2) Swiss Institute of Bioinformatics, University of Lausanne; 5) Bioinformatics and Biostatistics Core Facility, School of Life Sciences, Ecole Polytechnique Fédérale de Lausanne (EPFL); 2) Swiss Institute of Bioinformatics, University of Lausanne; 5) Bioinformatics and Biostatistics Core Facility, School of Life Sciences, Ecole Polytechnique Fédérale de Lausanne (EPFL); 3) The Institute of Bioengineering (IBI), School of Life Sciences, Ecole Polytechnique Fédérale de Lausanne (EPFL); 3) The Institute of Bioengineering (IBI), School of Life Sciences, Ecole Polytechnique Fédérale de Lausanne (EPFL); 2) Swiss Institute of Bioinformatics, University of Lausanne; 5) Bioinformatics and Biostatistics Core Facility, School of Life Sciences, Ecole Polytechnique Fédérale de Lausanne (EPFL); 1) Center for Integrative Genomics, Faculty of Biology and Medicine, University of Lausanne; 4) Vital IT, Swiss Institute of Bioinformatics; 3) The Institute of Bioengineering (IBI), School of Life Sciences, Ecole Polytechnique Fédérale de Lausanne (EPFL); 5) Bioinformatics and Biostatistics Core Facility, School of Life Sciences, Ecole Polytechnique Fédérale de Lausanne (EPFL); 3) The Institute of Bioengineering (IBI), School of Life Sciences, Ecole Polytechnique Fédérale de Lausanne (EPFL); 3) The Institute of Bioengineering (IBI), School of Life Sciences, Ecole Polytechnique Fédérale de Lausanne (EPFL); 4) Vital IT, Swiss Institute of Bioinformatics; 3) The Institute of Bioengineering (IBI), School of Life Sciences, Ecole Polytechnique Fédérale de Lausanne (EPFL); 5) Bioinformatics and Biostatistics Core Facility, School of Life Sciences, Ecole Polytechnique Fédérale de Lausanne (EPFL); 1) Center for Integrative Genomics, Faculty of Biology and Medicine, University of Lausanne; 2) Swiss Institute of Bioinformatics, University of Lausanne; 2) Swiss Institute of Bioinformatics, University of Lausanne; 7) Bioinformatics Core Facility, Swiss Institute of Bioinformatics; 8) Department of Oncology and Ludwig Center for Cancer Research, Faculty of Biology and Medicine, University of Lausanne; 6) Department of Molecular Biology, Faculty of Sciences, University of Geneva; 1) Center for Integrative Genomics, Faculty of Biology and Medicine, University of Lausanne; 1) Center for Integrative Genomics, Faculty of Biology and Medicine, University of Lausanne; 6) Department of Molecular Biology, Faculty of Sciences, University of Geneva; 1) Center for Integrative Genomics, Faculty of Biology and Medicine, University of Lausanne; 1) Center for Integrative Genomics, Faculty of Biology and Medicine, University of Lausanne; 3) The Institute of Bioengineering (IBI), School of Life Sciences, Ecole Polytechnique Fédérale de Lausanne (EPFL); 1 Center for Integrative Genomics, Faculty of Biology and Medicine, University of Lausanne, Lausanne, Switzerland; 2 Vital IT, Swiss Institute of Bioinformatics, Lausanne, Switzerland; 3 Department of Molecular Biology, University of Geneva, Geneva, Switzerland; Charité - Universitätsmedizin Berlin, Germany

## Abstract

In mammals, the circadian clock allows them to anticipate and adapt physiology around the 24 hours. Conversely, metabolism and food consumption regulate the internal clock, pointing the existence of an intricate relationship between nutrient state and circadian homeostasis that is far from being understood. The Sterol Regulatory Element Binding Protein 1 (SREBP1) is a key regulator of lipid homeostasis. Hepatic SREBP1 function is influenced by the nutrient-response cycle, but also by the circadian machinery. To systematically understand how the interplay of circadian clock and nutrient-driven rhythm regulates SREBP1 activity, we evaluated the genome-wide binding of SREBP1 to its targets throughout the day in C57BL/6 mice. The recruitment of SREBP1 to the DNA showed a highly circadian behaviour, with a maximum during the fed status. However, the temporal expression of SREBP1 targets was not always synchronized with its binding pattern. In particular, different expression phases were observed for SREBP1 target genes depending on their function, suggesting the involvement of other transcription factors in their regulation. Binding sites for Hepatocyte Nuclear Factor 4 (HNF4) were specifically enriched in the close proximity of SREBP1 peaks of genes, whose expression was shifted by about 8 hours with respect to SREBP1 binding. Thus, the cross-talk between hepatic HNF4 and SREBP1 may underlie the expression timing of this subgroup of SREBP1 targets. Interestingly, the proper temporal expression profile of these genes was dramatically changed in *Bmal1* ^−/−^ mice upon time-restricted feeding, for which a rhythmic, but slightly delayed, binding of SREBP1 was maintained. Collectively, our results show that besides the nutrient-driven regulation of SREBP1 nuclear translocation, a second layer of modulation of SREBP1 transcriptional activity, strongly dependent from the circadian clock, exists. This system allows us to fine tune the expression timing of SREBP1 target genes, thus helping to temporally separate the different physiological processes in which these genes are involved.

## Author Summary

Circadian rhythmicity is part of our innate behavior and controls many physiological processes, such as sleeping and waking, activity, neurotransmitter production and a number of metabolic pathways. In mammals, the central circadian pacemaker in the hypothalamus is entrained on a daily basis by environmental cues (i.e. light), thus setting the period length and synchronizing the rhythms of all cells in the body. In the last decades, numerous investigations have highlighted the importance of the internal timekeeping mechanism for maintenance of organism health and longevity. Indeed, the reciprocal regulation of circadian clock and metabolism is now commonly accepted, although still poorly understood at the molecular level. Our global analysis of DNA binding along the day of Sterol Regulatory Element Binding Protein 1 (SREBP1), a key regulator of lipid biosynthesis, represents the first tool to comprehensively explore how its activity is connected to circadian-driven regulatory events. We show that the regulation of SREBP1 action by nutrients relies mainly on the control of its subcellular localization, while the circadian clock influences the promoter specific activity of SREBP1 within the nucleus. Furthermore, we identify the Hepatocyte Nuclear Factor 4 (HNF4) as a putative player in the cross-talk between molecular clock and metabolic regulation.

## Introduction

Mammals possess an internal circadian clock which allows them to anticipate and adapt to daily environmental changes [1]. The molecular mechanism underlying the cell-autonomous circadian rhythms relies on a network of feedback loops in which BMAL1, CLOCK, Neuronal PAS domain (NPAS) protein 2 and Retinoic acid receptor-related Orphan Receptor (ROR) proteins act as transcriptional activators and period homolog proteins (PER1, 2 and 3), cryptochromes (CRY1 and 2) and REV-ERBs function as inhibitors producing the self-sustained oscillating production of their target genes, including themselves. At central level, the expression of clock genes is dictated by a pacemaker localized in the hypothalamic suprachiasmatic nucleus (SCN) which synchronizes the phase in nearly all body cells. However, in peripheral organs such as the liver, oscillations are also entrained by the feeding and fasting cycle [2], [3], [4]. This sophisticated regulatory system contributes to coordinate many physiological processes, such as sleep-wake cycles, locomotor activity, body temperature, hormone secretion and energy metabolism that all display circadian rhythms. In particular, the importance of the connection between circadian clock and metabolism regulation is emerging. Epidemiological studies have shown an increased incidence of obesity, diabetes, and cardiovascular disease, in addition to certain cancers and inflammatory disorders in night workers [5]–[7]. Accordingly, in genetic mouse models the disruption of the clock alters metabolic homeostasis at different levels (reviewed in [8]), suggesting a still unresolved relationship between nutrient state and circadian homeostasis.

The Sterol Regulatory Element Binding Protein 1 (SREBP1), a basic Helix-Loop-Helix-Leucine Zipper (bHLH-LZ) transcription factor, plays a key role in the regulation of lipid biosynthesis, which is one of the most feeding-related function in the liver [9]. SREBP1 is synthesized as an inactive precursor, anchored to the ER-membrane and its N-terminal fragment is released into the nucleus after proteolytic cleavage in response to cholesterol depletion [10] or to activation of the insulin signalling pathway [11], [12]. Two SREBP1 isoforms, 1a and 1c, are obtained through alternative splicing of the same gene [13], [14]. The liver expresses mostly the SREBP1c isoform that mediates the insulin-driven lipogenic activity [15]. Besides being under the control of the feeding-fasting cycle, SREBP1 translocation to the nucleus is also influenced by one of the master clock regulators, REV-ERBα [16]. Nevertheless, in absence of a functional clock, such as in *cry1^−/−^;cry2^−/−^* mice, a normal expression pattern of several SREBP1 target genes can be restored by an imposed rhythmic food intake [4], suggesting a dominant role of the feeding-fasting cycle in the regulation of SREBP1.

To systematically understand how the interplay of circadian clock and nutrient-driven rhythm regulate SREBP1 activity, we evaluated the genome-wide binding of SREBP1 to its targets along the day in wild-type mice. Our results define SREBP1 binding pattern in the physiological context of both rhythmic food absorption and circadian rhythm and they give the first tools to comprehensively explore how SREBP1 activity is connected to circadian-driven regulatory events.

## Results

### SREBP1 binding to DNA is rhythmic

**Figure 1:**
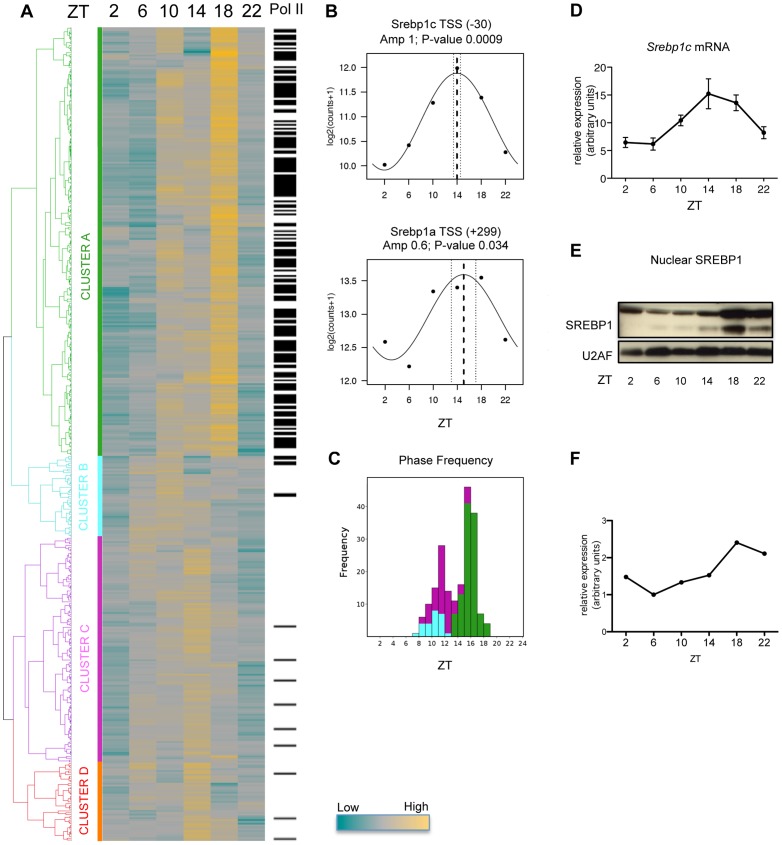
Dynamics of SREBP1 binding. (A) C57BL/6 mice were fed only during the night (ZT12-ZT24) for one week before collecting liver every 4 hours for one day. Chromatin from 5 mice was pooled at each time point and ChIP with an antibody against SREBP1 was performed. Peaks were positioned where the signal for SREBP1 was at least a four-fold in comparison to the input signal in at least one time point. The heat-map represents SREBP1 binding to all its targets along the time. Hierarchical clustering was done using Pearson correlation scores and identified four major clusters (A, B, C and D). The color scale is indicated below. In the column on the right, black lines indicate that Pol II was detected in the same site as SREBP1 in at least one time point, as assessed in our previous ChIP-seq data set [24]. (B) Two SREBP1 binding sites were identified in the proximity of *Srebp1* gene, at a distance of −30 and +299 nucleotides from the *Srebp1c* and *Srebp1a* TSS, respectively. Graphs represent the fitting to a cosine function of experimental data obtained on these peaks (black dots), in order to calculate the phase of the binding (dashed line), its interval of confidence (dotted lines) and the associated P-value. (C) Histogram of binding phase frequency in clusters A, B and C, for peaks with a P-value of the amplitude <0.1. None of the peaks belonging to cluster D met this requirement. (D) mRNA expression of *Srebp1c* was evaluated by qPCR in livers from C57BL/6 mice at the indicated ZT time (n = 5). Data are normalized using 36B4 as housekeeping gene. (E) Hepatic nuclear extracts from C57BL/6 mice were subjected to western blot analysis to detect the nuclear SREBP1. U2AF was used as loading control. Each sample is a pool of 5 livers. (F) Quantification of the Western Blot was performed by densitometry, using ImageJ software.

To evaluate the genome-wide dynamics of SREBP1 binding to its target sites in a physiological context, we prepared liver chromatin from C57BL/6 mice, collecting samples each 4 hours during one day (see Material and Methods). ChIP-seq with an antibody that recognizes both SREBP1 isoforms was performed at each time point. The SREBP1 antibody was tested extensively (Figure S1) and has also been used in previous studies [17]. We obtained an average of 38 millions sequence reads by time point by ultra-high-throughput sequencing (Table S1). The mapping allowed the identification of 448 bona fide SREBP1 binding peaks, above the background. As shown in Figure 1A, the binding of SREBP1 is overall oscillatory, with the maximum for most of the sites at Zeitgeber Times (ZT) 14 or 18 (light is on at ZT0 and is off at ZT12). To systematically evaluate the rhythmicity of SREBP1 recruitment to its targets, a cosine function was fitted to the temporal profile of the binding (see Material and Methods). This allowed to calculate, for each peak, the binding phase and the amplitude of the oscillation together with its associated P-value, as exemplified for the two sites found on the *Srebp1* gene itself (Figure 1B). 53% of SREBP1 binding sites were found to be rhythmic (P<0.1 for the amplitude). Four clusters of targets were clearly distinguishable based on binding kinetics (Figure 1A), the first with a phase distributed around ZT15–ZT17, whereas the other ones with the phase peaking around ZT11–ZT12, as determined by cosine function (Figure 1C). The observed kinetics of SREBP1 binding, especially for cluster A peaks, was consistent with its gene expression and nuclear localization, (Figure 1D, 1E and 1F). Collectively, these results show that the activity of SREBP1 oscillates with a pronounced circadian rhythm, in agreement with the previously reported daily variations of its RNA and protein levels [16], [18]–[20].

### Characterization of SREBP1 binding sites

**Figure 2:**
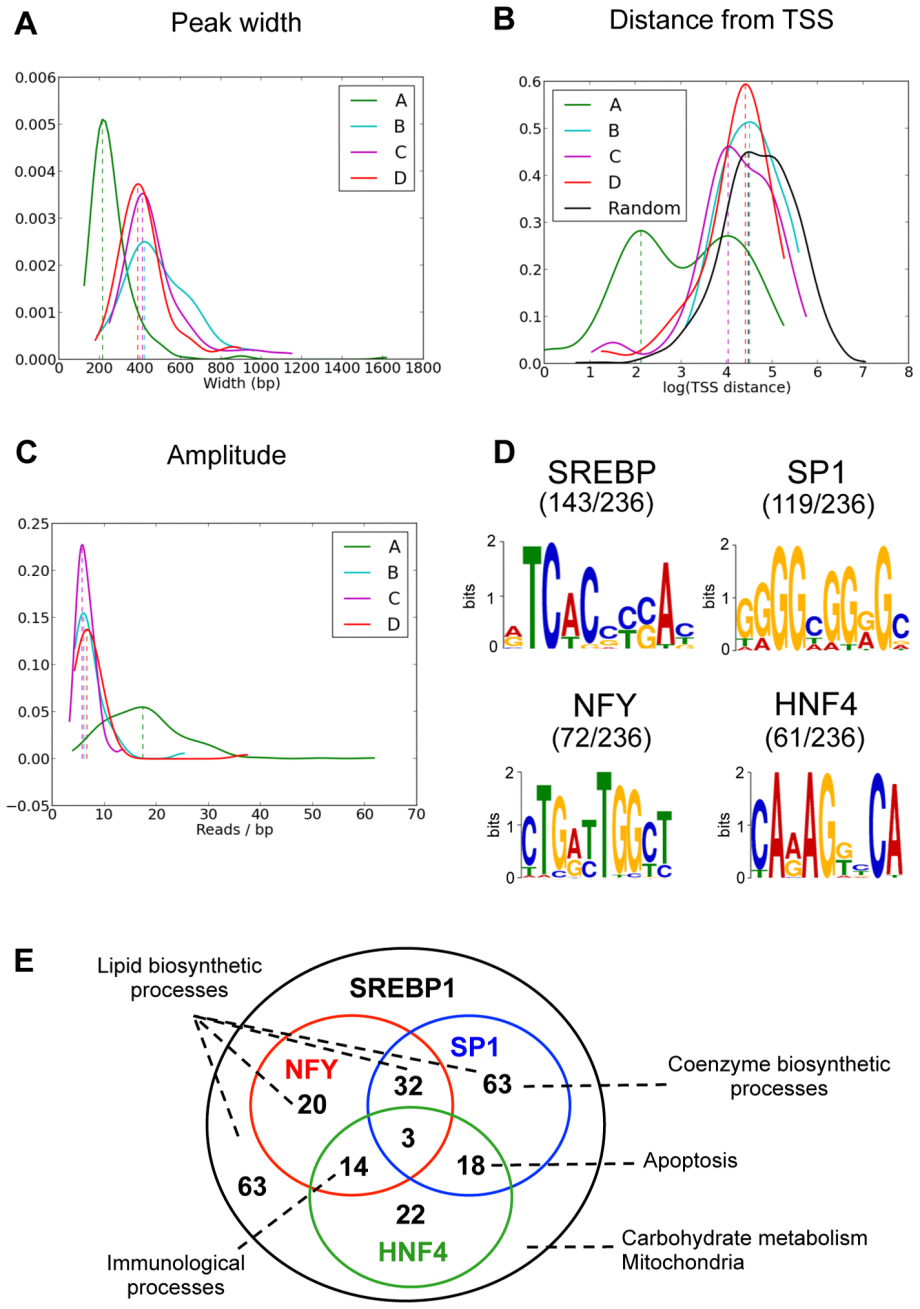
Features of SREBP1 binding sites. (A) Distribution of peak lengths in the four clusters of SREBP1 binding sites shown in figure 1A. (B) Distribution of the distance from the closest annotated TSS of SREBP1 binding peaks belonging to the four clusters. Cluster A is enriched in sequences closer to a TSS, whereas the profile of cluster B, C and D is overlapping with that of 1000 randomly selected sequences (black line). (C) Distribution of the amplitudes of SREBP1 binding oscillation. (D) Overrepresented motifs within SREBP1 binding sites belonging to cluster A were found using MEME [21]. DNA sequences in the window under each SREBP1 peak were used for the sequence analysis. (E) The 236 SREBP1 binding sites (distributed in 223 genes) belonging to cluster A were associated to the closest genes. Annotations are available for 219 of these 224 genes and the Venn diagram shows the overlap between the presence of SREBP1 and the presence of a nearby a binding site for SP1, NFY and/or HNF4. The most relevant functional pathways that were enriched in the different sets of target genes are indicated.

SREBP1 binding sites identified in this study are grouped in four clusters with a slightly shifted phase. To better investigate the features of these sites we calculated the number of nucleotides spanned by each peak and found that sites belonging to cluster A (236 out of 448) were narrow, with a typical length of about 200 nucleotides (Figure 2A). These peaks were also closer to the nearest annotated transcription start site (TSS) than peaks belonging to clusters B, C or D, whose distance to the nearest annotated TSS roughly matches randomly picked genomic locations (Figure 2B). Moreover, the amplitude of the binding oscillation along the 24 hours was greater for cluster A peaks (Figure 2C). These observations suggest that cluster A sites may be more relevant in the regulation of transcription mediated by SREBP1. In agreement with this hypothesis, a MEME [21] motif search analysis clearly identified the canonical SREBP1 consensus motif in more than 60% of the sites belonging to cluster A (Figure 2D), but only in 6% of the sites assigned to clusters B, C and D. Within cluster A, the MEME analysis also showed that motifs for SP1 and NFY, two transcription factors (TFs) known to cooperate with SREBP1 to regulate the transcription of its target genes, were overrepresented [17]. The consensus motif for the Hepatic Nuclear Factor 4 (HNF4) was also identified in 61 out of the 236 cluster A sites. The discovered motifs were enriched in cluster A peaks with an empirical p-value<0.001, as shown in Table S2. In addition, in the regions belonging to cluster B, C and D, we determined only several highly repetitive sequences as top-scoring motifs (for example ACACACACA in 73 sites out of 212) that could not be associated to any known consensus motif for TFs. This result suggests that SP1, NFY and HNF4 may participate to SREBP1-mediated transcriptional regulation and further supports the functional importance of SREBP1 binding sites assigned to cluster A. Thus, we opted to focus the following analyses on these regions, although we cannot exclude that the other sites might contribute to mediate SREBP1 activity in mouse liver potentially through genome loops.

To explore the cellular processes that are regulated by SREBP1 along the day, we annotated each site with the nearest Ensembl transcript. We used DAVID [22], [23] to identify clusters of genes enriched with functional annotations. As expected, we identified lipid biosynthetic processes and fatty acid metabolism as the most prominent pathways controlled by SREBP1 (Table S3). In addition, we found a significant enrichment of genes involved in carbohydrate metabolism, in the response to nutrient levels, in mitochondrial and endoplasmic reticulum functions and in coenzyme metabolism. In a previous genome-wide study performed in human HepG2 cells, it was shown that unique combinations of SREBP1, SP1 and NFY target distinct functional pathways [17]. Since we found a good enrichment within the SREBP1 binding sites of the consensus motifs for NFY and SP1, but also HNF4, we explored whether a network among these three transcription factors could be highlighted in mouse liver. As shown in Table 1, genes involved in lipid biosynthesis and in the regulation of fatty acid and steroid metabolism were highly represented in all categories. In some cases, however, one biological function was targeted by a unique combination of regulators. For example, the biosynthesis of coenzymes was selectively represented within the genes bearing only the site for SP1, whereas both SP1 and HNF4 motifs were present in genes involved in apoptosis. Likewise, a combination of HNF4 and NFY motifs marked most of the genes involved in immunological processes. Finally, pathways related to carbohydrate metabolism and mitochondria were particularly enriched in genes without NFY or SP1 motifs, suggesting that SREBP1 may cooperate with other regulators at the promoter of these genes (the complete functional annotation clustering is reported in Table S4). Our results suggest that in mouse liver, in physiological conditions, the network SREBP1-SP1-NFY-HNF4 may be important in order to determine the functional effect of SREBP1 binding (Figure 2E).

**Table 1:** Functional annotation clustering of putative SREBP1 targets using DAVID tools.

Binding Motif	n	Functional cluster	GO Terms	P-Value
SP1	65(63)	sterol metabolism	GO:0016125	6.50E-07
		coenzyme biosynthesis	GO:0009108	1.40E-03
		metal ion binding	GO:0046872	6.30E-04
		lipid biosynthesis	GO:0008610	4.20E-04
		magnesium ion binding	GO:0000287	4.20E-05
		cell fractions	GO:0000267	0.029
NFY	21(20)	lipid biosynthesis	GO:0008610	2.70E-06
HNF4	22(22)	No clusters		
SP1+NFY	32(32)	nucleotide binding	GO:0005524	2.70E-03
		sterol metabolism	GO:0016126	9.60E-04
		inflammatory response	GO:0006954	0.04
SP1+HNF4	18(18)	cholesterol metabolism	GO:0008203	2.80E-05
		regulation of apoptosis	GO:0042981	0.033
NFY+HNF4	15(14)	antigen processing….	GO:0002474	6.60E-05
SP1+NFY+HNF4	4(3)	No clusters		
Only SREBP1	65(63)	glycerolipid metabolism	GO:0046486	9.40E-03
		fatty acid metabolism	GO:0006631	0.024
		glucose metabolism	GO:0006006	0.041
		mitochondria	GO:0005739	5.00E-03

Enriched GO categories were identified in four distinct sets of SREBP1 target genes exhibiting a different combination of binding sites for SP1, NFY and/or HNF4. In total, 219 out of 223 SREBP1 putative target genes have a functional annotation (the number of annotated genes for each set is indicated in parentheses). The analysis using DAVID groups the GO categories in functional related clusters. For each enriched cellular process, only the most significant associated GO term is shown in the table, with the corresponding Modified Fisher Exact P-value. The complete list of all GO terms enriched in each functional cluster for all the groups is available in Table S4.

### RNA polymerase II recruitment to SREBP1 target genes is not always synchronized with SREBP1 binding

**Figure 3:**
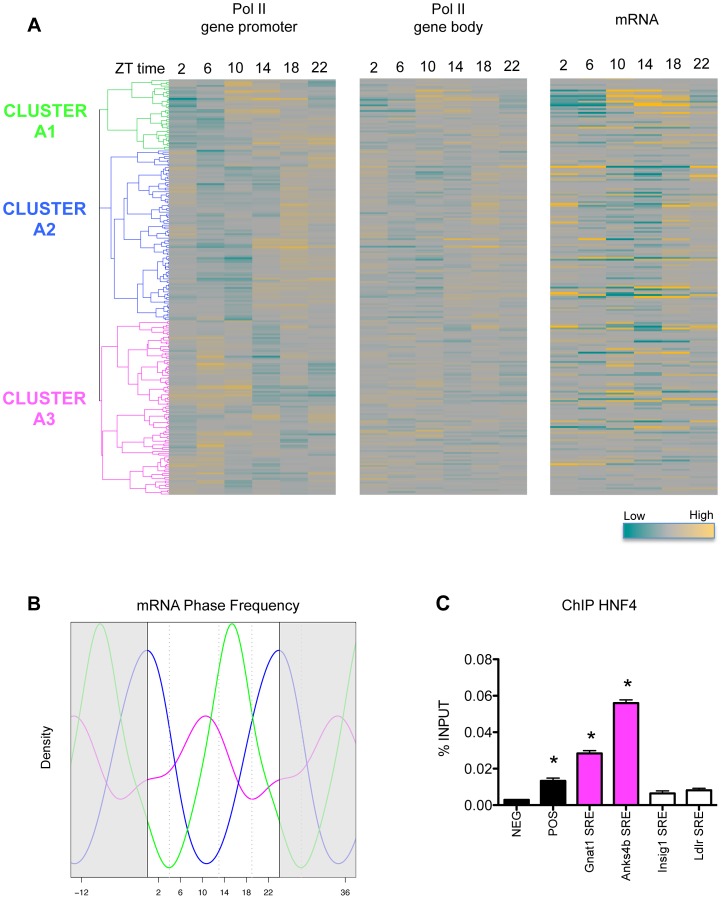
Pol II recruitment on SREBP1 target genes is not always synchronized with SREBP1 binding. (A) The heat-maps represent the recruitment of Pol II to the promoter (left) and to the gene body (middle) of SREBP1 putative target genes along the day, as assessed in our previous ChIP-seq data set [24]. In parallel, we evaluated hepatic gene expression by microarray analysis in the same samples (right). Hierarchical clustering was done applying a Pearson correlation scores to the data describing Pol II recruitment on the promoters of SREBP1 target genes (left). Three major clusters of genes displaying a different temporal binding profile of Pol II were identified (A1, A2, and A3). The genes are ordered in the three heat-maps according to this clustering. (B) Gene expression data from microarray analysis were fit to a cosine function to estimate the phase of expression (peak time of the fit) of SREBP1 target genes. The graph shows the smoothing of phase distributions of the genes belonging to the three clusters (green line for A1, blue line for A2, magenta line for A3). Only genes with a P-value<0.05 are plotted. Dotted lines define three time intervals containing the most recurrent phases associated to the genes belonging to the clusters A1, A2 and A3. (C) HNF4 binding was tested on randomly selected SRE identified in our SREBP1 ChIP-seq. *Gnat1* and *Anks4b* SREs belong to cluster A3 and contain a HNF4 putative binding sites. In contrast, *Ldlr* and *Insig1* SREs do not contain in their sequence a HNF4 motif and belong to cluster A2. NEG and POS were used as negative and positive control loci and correspond to two regions of *Cyp7a1* promoter, localized at −1500 and −150 from the TSS, respectively [69]. The graph shows the mean ± SEM of three independent experiments. * indicates P-value<0.01 vs. NEG. Statistical analysis was performed by one-way ANOVA followed by Bonferroni post-test. Primer sequences are listed in Table S8.

In Figure 1, we showed that SREBP1 binds to target sites belonging to cluster A with a sharp phase between ZT15 and ZT17. To investigate the functional effects of SREBP1 binding on gene transcription, we checked in our previously reported data set [24] the 24 hours profile of RNA polymerase II (Pol II) recruitment in the proximity of SREBP1 target genes. Importantly, most of the SREBP1 binding sites belonging to cluster A were co-occupied by Pol II (Figure 1A), further supporting the functional relevance of these regions. We next evaluated Pol II binding to the promoter and in the gene body of all putative SREBP1 target genes. In parallel, we measured mRNA levels of the same genes by microarray analysis (Table S5). More than 85% of SREBP1 target genes show an expression level above the median expression level of all the transcripts, suggesting that they are transcribed. Our analyses revealed three clusters of target genes, that we called A1, A2 and A3, with distinct temporal profile of transcription and expression (Figure 3A and Figure S2). In cluster A1, the peak of Pol II binding was concomitant, or even slightly earlier than SREBP1 binding. In contrast, for genes belonging to cluster A2, Pol II association to both promoter and gene body strictly followed SREBP1 binding. Lastly, Pol II recruitment to the genes of the A3 group was shifted by about +8 h with respect to SREBP1. For all clusters, the temporal profile of gene expression was consistent with the dynamics of Pol II association. The distribution of all the expression phases obtained for the genes belonging to the three groups confirmed that SREBP1 target genes are expressed in different moments of the day, in spite of the concomitant binding of the transcription factor (Figure 3B). This observation suggests that other factors participate in the regulation of the various SREBP1 target genes in order to assure their appropriate expression timing. Interestingly, genes that were mainly expressed during the fed state (clusters A1 and A2) were functionally enriched in the regulation of lipid and coenzyme biosynthetic processes, as well as in the response to hormones, such as insulin. In contrast, SREBP1 target genes involved in mitochondrial oxidation and apoptosis were enriched during the fasting period (Table 2). Thus, the promoter specific events that determine the different temporal expression profile of SREBP1 target genes contribute to define the set of cellular functions that are active at a given time.

**Table 2:** Functional annotation clustering of putative SREBP1 targets with a different temporal expression profile.

PHASE	n	Functional cluster	GO term	P-value
ZT13-ZT19	42	cholesterol metabolism	GO:0008203	0.01
		amino acid binding	GO:0016597	2.00E-03
		response to starvation	GO:0042594	1.90E-03
		response to hormones	GO:0009725	6.10E-03
		glycerolipid metabolism	GO:0046486	3.10E-03
		cell migration	GO:0016477	0.017
ZT19-ZT28.4	68	sterol metabolism	GO:0016126	1.60E-09
		magnesium ion binding	GO:0000287	1.00E-03
		coenzyme biosynthesis	GO:0006732	0.019
		nucleotide binding	GO:0032555	0.041
		endoplasmic reticulum	GO:0005783	0.041
ZT4.4-ZT13	39	mitochondria	GO:0005739	3.20E-03
		regulation of apoptosis	GO:0042981	6.70E-03
		regulation of myeloid cell differentiation	GO:0045637	2.30E-03

Enriched GO categories were identified in three distinct sets of rhythmic SREBP1 target genes (P<0.05), based of their phase of expression. To define the three intervals of time we calculated the shortest time range containing the phases of at least 50% of the genes belonging to clusters A1, A2 or A3. For each set, the total number of genes is indicated and the number of genes with annotation is indicated in parentheses. For each functional cluster, only the most significant associated GO term is shown in the table, with the corresponding Modified Fisher Exact P-value.

### A functional core molecular clock is necessary for the timing of SREBP1 target gene expression

**Figure 4:**
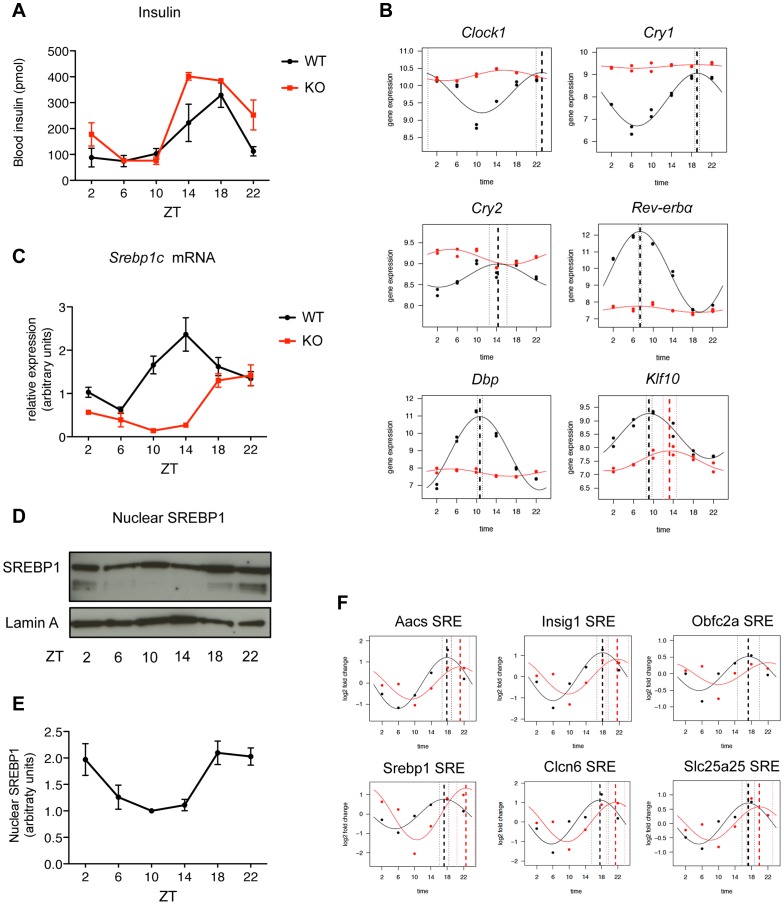
SREBP1 binding is rhythmic in *Bmal1^−/−^* upon time-restricted feeding. (A) *Bmal1^−/−^* (red line) and control mice (black line) were fed only during the night for one week before the sacrifice. Plasma insulin levels were measured at the indicated time points (n = 3–6). (B) mRNA levels of key genes of the cellular molecular clock were measured by qPCR in *Bmal1^−/−^* and control mice (n = 5). (C) Hepatic expression of *Srebp1c* was evaluated by qPCR in *Bmal1^−/−^* and control mice (n = 5). Data are normalized using *36b4* and *Rps9* as housekeeping genes. (D) Representative western blot analysis of the nuclear SREBP1 in hepatic nuclear extracts from *Bmal1^−/−^* mice. Lamin A was used as loading control. Each sample is a pool of 5 livers. (E) Western Blot quantification was performed by densitometry, using ImageJ software. Each point represents the mean ± SEM of the quantification of three analyses performed in three independent sets of *Bmal1^−/−^* mice (n≥3 per each time point). (F) ChIP of SREBP1 was performed in livers of *Bmal1^−/−^* and control mice at the indicated time points. SREBP1 binding was tested on 6 loci (SRE) identified by ChIP-seq in the proximity of the indicated genes. *Aacs* and *Srebp1c* belong to cluster A1, *Insig1* and *Clcn6* belong to cluster A2, whereas *Obfc2a* and *Slc25a25* belong to cluster A3. Primer sequences used for qPCR analyses are available in Tables S7 and S8.

**Figure 5:**
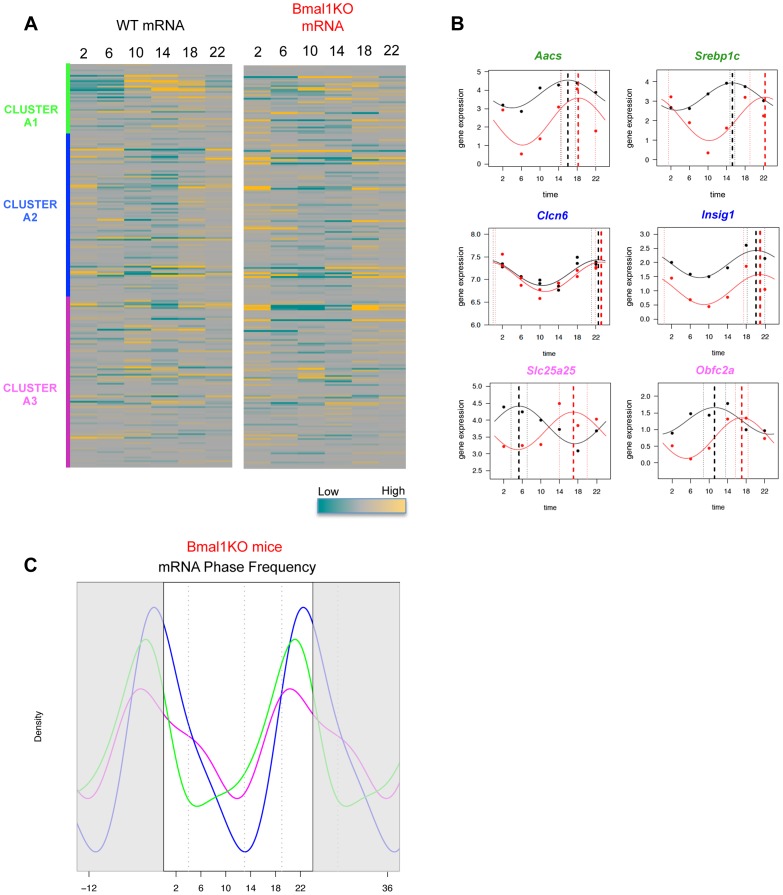
Phase of expression of rhythmic SREBP1 target genes is perturbed in *Bmal1^−/−^* upon time-restricted feeding. (A) The heat-maps represent the hepatic gene expression of putative SREBP1 target genes in wild type (left) and *Bmal1^−/−^* mice (right), after one week of time-restricted feeding, as assessed by microarray analysis. The order of the genes is based on the clustering shown in figure 3 and is maintained in the two heat-maps. (B) Validation of hepatic mRNA level variation of a panel of the indicated SREBP1 putative target genes. (C) Gene expression data from microarray analysis in *Bmal1^−/−^* mice were fit to a cosine function to estimate the phase of expression (peak time of the fit) of SREBP1 target genes. The graph shows the smoothing of phase distributions of the genes belonging to the three clusters (green line for A1, blue line for A2, magenta line for A3). Only genes showing a P value<0.05 in *Bmal1^−/−^* mice are plotted.

To understand the molecular mechanism underlying the different temporal expression of SREBP1 target genes, we first explored the possible involvement of the network SREBP1-SP1-NFY-HNF4 in determining the functional effect of the binding of SREBP1 to its targets. To check for the presence of a pattern characterizing the three groups of SREBP1 target genes identified earlier (see Figure 3), we evaluated the presence of different combinations of SP1 and NFY motifs and their orientation with respect to the SREBP1 binding sites (data not shown). However, we could not establish any significant correlation. In contrast, we found that HNF4 motifs were significantly overrepresented (P-value<0.02) in the regions under SREBP1 peaks of the genes expressed during the fasting period (cluster A3), compared to the other clusters (Table S6). The actual recruitment of HNF4 to these putative binding sites was assessed by ChIP on randomly selected SREBP1 Responsive Elements (SREs) (Figure 3C). Besides HNF4, other transcription factors, such as the cAMP response element-binding protein (CREB) or Forkhead box proteins O (Foxo), are important players in the hepatic metabolic regulation upon fasting [25], [26]. However, their known consensus motifs were not found in the proximity of cluster A3 SREBP1 peaks. These observations strongly suggest a specific cross-talk between HNF4 and SREBP1 in the regulation of these genes. To further investigate which control processes dictate the distribution of SREBP1 target gene expression along the day, we then considered the possible role of the circadian rhythm in this regulation. To test this hypothesis, it was necessary to uncouple the circadian rhythm from the response to nutrients. Thus, we fed mice lacking BMAL1 (*Bmal1* ^−/−^) only during the darkness period for one week before collecting liver samples every four hours. Upon this experimental conditions, the circadian clock was completely disrupted in *Bmal1^−/−^* mice, as demonstrated by the flattened expression of key core and output components of the clock, such as *Clock1*, *Cry1*, *Cry2*, D site albumin promoter binding protein (*Dbp*), *Rev-Erbα* and Kruppel-like factor 10 (*Klf10*) (Figure 4B). Body weight, daily food intake and glycemia were unchanged in *Bmal1* ^−/−^ mice (Figure S3). Importantly, the imposed rhythmic food intake restored an oscillatory nutrient response, as shown by the levels of circulating insulin that were comparable to the wild type (Figure 4A). Accordingly, in *Bmal1^−/−^* mice SREBP1 translocated to the nucleus from ZT18 onwards (Figure 4D and 4E) and its expression was still cycling, although the phase was delayed by about 6 h compared to the wild type (Figure 4C). We next checked the dynamics of SREBP1 binding to a panel of the targets previously identified in wild type mice and we found that SREBP1 was recruited to all the tested sites in an oscillatory way, but with an average phase shift of about 4 hours (figure 4F). Finally, to assess the impact of the circadian oscillator impairment on SREBP1-driven transcription, we globally evaluated the expression of SREBP1 target genes in *Bmal1^−/−^* mice (Table S5). Most SREBP1 target genes were still scored as oscillating in *Bmal1^−/−^* upon temporal restricted feeding (57% have a P<0.05). However, the heatmap rendering of their expression patterns (Figure 5A) revealed a temporal profile that was perturbed in *Bmal1^−/−^* compared to WT mice, as most of the genes now had a maximum expression at ZT18, coinciding with the binding of the transcription factor (Figure 5C). Accordingly, the expression phases of the genes belonging to the clusters A1 and A3 identified earlier (Figure 3) were now largely concomitant with those of genes belonging to cluster A2, therefore mostly grouped between ZT14 and ZT24 (Figure 5B and Figure S4). This phase shift was not due to a selective decrease of the number of cycling genes in clusters A1 and A3, as the percentage of the significantly oscillating genes was comparable in the three clusters in WT and *Bmal1^−/−^* mice (cluster A1: 84% in WT vs 94% in *Bmal1^−/−^*; cluster A2: 71% in WT vs 66% in *Bmal1^−/−^*; cluster A3: 62% in WT vs 64% in *Bmal1^−/−^* with P<0.05).

To explore how the core clock components participate to this regulation we checked in published data sets whether key transcription factors such as BMAL1, CLOCK1, CRY1, CRY2, PER1, PER2, NPAS2 and REV-ERBs can differentially bind to the promoters of the three clusters of SREBP1 target genes [27]–[29]. Interestingly, we found that most SREBP1 peaks (148 out of 236, ≈63%) have an overlapping REV-ERBα and/or REV-ERBβ peak. In addition, a REV-ERBα/β binding site was detected also in another 17% of the promoters of SREBP1 target genes, but in a non-overlapping position. This strong occupancy of SREBP1 targets by REV-ERBs is consistent with the previously reported involvement of REV-ERBα in the regulation of lipid metabolic genes [30], and suggests the existence, in mouse liver, of a SREBP1-REV-ERBs network in physiological conditions. The frequency of REV-ERBs recruitment was comparable in clusters A1, A2 and A3 (data not shown), thus arguing against the possible role of these nuclear receptors in determining the distinct phase of expression of these genes. However, due to the presence of REV-ERB binding sites in many SREBP1 target genes, the flattened REV-ERB expression observed in *Bmal1^−/−^* may perturb, at least in part, the phase of several SREBP1 target genes. Indeed, in WT mice, the temporal expression profile of SREBP1 and REV-ERBs is very different (the phases of expression are ZT15 and ZT8, respectively [16]), and these factors are not expected to compete for binding at the same time to the same genes. The other transcription factors tested were recruited to a lesser extent on SREBP1 target gene promoters and for none of them we observed a significant enrichment in clusters A1, A2 or A3 (data not shown).

Taken together, our results confirm that SREBP1 activity is strongly dictated by the rhythmicity of nutrient intake. In addition, our observations indicate that a functional circadian core clock is necessary to assure the correct temporal expression profile of SREBP1 target genes and suggest a role for HNF4 in dictating the phase of expression of genes whose mRNA levels peak when SREBP1 binding is low. Further studies will aim at understanding whether and how the lack of circadian rhythm perturbs HNF4 activity.

## Discussion

SREBP1 is a highly circadian transcription factor whose activity is strongly regulated by nutrient availability through the insulin signaling pathway. In mouse liver SREBP1 expression displays a daily rhythm with a peak in the nocturnal feeding period under standard housing condition of mice [16], [18]–[20]. In this study we evaluate the dynamics of SREBP1 recruitment to DNA by determining its genome wide *cis*-acting targets (cistrome) in the liver along an entire day. SREBP1 binds to 448 sites with an oscillatory profile that is temporally coherent with the phase of its maximal expression. Within SREBP1 binding sites, four distinct groups are clearly distinguishable. The first set (cluster A) contains peaks that are likely the more relevant in the transcriptional regulation mediated by SREBP1 as they are the closest to TSS and they are bound more rhythmically by SREBP1. Importantly, in more than 60% of these sites we identified the direct repeat 5′-ATCACCCCAC-3′ that was described as the Sterol regulatory proteins Responsive Elements (SRE) in several promoters, such as the human LDL receptor promoter [13], [31]–[33]. This direct repeat variant of the canonical E-box inverted repeats 5′-CAnnTG-3′ was shown to be specifically recognized by SREBP proteins due to the presence of a tyrosine residue in a position that corresponds to an arginine in all the other bHLH-LZ proteins and that is critical for high affinity contacts with the SRE [34], [35]. Furthermore, our ChIP-seq results highlighted the presence of predicted binding sites for SP1, NFY and HNF4 in 60%, 30% and 25% of cluster A sites, respectively. Since SREBP1c, the major SREBP1 isoform in the liver, is a weak transcriptional activator, this observation is consistent with earlier studies demonstrating that the transcription factors SP1, NFY and CREB cooperate to regulate different SREBP1-responsive promoters [36]–[39]. In 2009, Seo *et al* have published a list of liver SREBP1 target genes obtained from a genome-wide study of mice subjected to 24 hours fasting followed by 12 hours refeeding with high carbohydrate diet [40], thus creating a condition where a very high SREBP1c activity is expected. In this study, a functional variant of the direct repeat SRE (5′ACTACANNTCCC-3′) was identified as a preferred site for SREBP1 binding, and no enrichment of the predicted NFY binding site was identified. The difference between this data set and ours can most likely be attributed to the difference in the specific experimental conditions, acute challenge on the one hand and physiological condition on the other hand (present study). Consistent with our study, both SP1 and NFY proteins were recruited on more than 30% of SREBP1 target genes in a genome-wide analysis of SREBP1 binding in HepG2 cell line [17]. In addition to SP1 and NFY, here we identify HNF4 as an important player of the interconnected regulatory circuit that may assure the specific regulation of SREBP1 target genes with distinct functions. Interestingly, consensus motifs for SP1, NFY and HNF4 were found to be overrepresented in the promoters of cycling genes in the liver [41], [42]. Since ≈70% of SREBP1 targets show a circadian gene expression, our results are in line with these bioinformatics predictions, supporting the involvement of these transcription factors in the complex transcriptional regulation of circadian rhythm in liver. The second set of SREBP1 target sites falls in the three clusters B, C and D. These peaks are not enriched in regions proximal to TSSs for mapped genes, nor in predicted motifs for known transcription factors. Furthermore the temporal profile of SREBP1 binding to these sites is flattened compared to the first set of SREBP1 target sites (cluster A). Additional studies are required to understand whether these peaks have a functional role, or whether they are bound in a secondary manner by SREBP1 due to the formation of DNA loops.

In the liver the expression of several known SREBP1c target genes is decreased in fasted mice, when the levels of SREBP1 are very low and increased upon refeeding, when both SREBP1 expression and nuclear translocation are induced [40], [43]. Accordingly, our analysis of Pol II recruitment on SREBP1 putative target genes, coupled with the measurement of their mRNA levels, revealed a maximum of transcription and expression during the fed state, namely between ZT12 and ZT24, for the majority of these genes. This is consistent with the binding of SREBP1 to DNA, which is higher at this time of the day. Nevertheless, a large set of SREBP1 target genes (cluster A3) displays a temporal expression profile strongly shifted with respect to SREBP1 recruitment. However, the different phase of expression observed in these genes is coherent with the dynamics of Pol II association to their promoter and gene body, thus arguing against a major involvement of post-transcriptional mechanisms in this delay and suggesting the existence of promoter specific events that determine the different temporal expression profile of SREBP1 target genes. In particular, the low level of expression of genes belonging to cluster A3 when SREBP1 is bound raises the question whether SREBP1 itself, or through interaction with coregulatory proteins, can act as transcriptional repressor for these genes. Interestingly, it was proposed that SREBP1 may act as negative regulator of the cytosolic phosphoenolpyruvate carboxykinase (*Pck-1*) gene by impairing the recruitment of the transcriptional coactivator Peroxisome Proliferator-Activated Receptors γ Coactivator -1 (PGC-1) on HNF4α [44]. To explain the negative effect of SREBP1 on this gene, a second mechanism was put forward by Chakravarty and colleagues, suggesting an interference between the binding of SREBP1 and SP1, due to the orientation on the opposite DNA strands of the two binding sites [45]. Although we find an enrichment of motifs for NFY and SP1 in the close proximity of SREBP1 peaks from the first set of target sites (clusters A1, A2 and A3), we do not observe a different presence and/or orientation of these sites with respect to SREBP1 peaks, among these three groups of target sites. Conversely, the high frequency of the HNF4 motif in the cluster A3 suggests that the cross-talk between HNF4 and SREBP1 may be a general mechanism through which SREBP1 negatively affects the transcription of a sub-set of its target genes. In agreement with this hypothesis, among the targets of SREBP1 expressed upon fasting we detect the Peroxisome Proliferator-Activated Receptor α (*Pparα*) gene, that was shown to be crucially regulated by HNF4 [46].

In recent years, growing evidences have highlighted the impact of circadian gene networks on nutrient balance and, on the other hand, the regulation of the circadian clock by metabolism and food consumption [47], [48]. Thus, circadian clock and metabolism converge in numerous ways to control the activity of a number of transcription factors that are essential for maintaining metabolic homeostasis, although the exact contribution of each input remains to be deciphered. Several studies demonstrated that upon restricted feeding (RF), namely when time and duration of food availability is limited in time, mice adjust to the feeding period within a few days, they display food anticipatory behavior and consume their daily food intake during that limited time [49]–[52]. This feeding regimen drives rhythms in arrhythmic and clock mutant mice or in animals with SNC ablations, thus uncoupling the circadian clock, synchronized by SCN, from the periphery [3], [4]. Many physiological activities that are normally dictated by the SCN master clock, such as hepatic P450 activity, body temperature, locomotor activity, and heart rate, are restored by RF. In the liver of *cry1^−/−^;cry2^−/−^* mice, RF restores the oscillatory circadian expression profile of a number of “feeding driven” transcripts, although with a small delay in their phase of expression, showing that the circadian clock anticipates changes in the feeding state and accelerates the transcriptional response to an acute activation or repression by feeding [4]. This is consistent with our observation that in *Bmal1^−/−^* mice, rhythmic SREBP1 expression and activity, that are drastically flattened when mice are fed *ad libitum* (data not shown), are reinstated upon RF, although with a deferred phase. Notably, growth and metabolic defects that were reported in *Bmal1^−/−^* mice either at older age or under different feeding regimens [53]–[58], are negligible in the experimental conditions adopted in our study, suggesting that the role of the circadian clock in the regulation of SREBP1 could be evaluated in the absence of major confounding pathologies. As an example, *Bmal1^−/−^* mice at 8–10 month of age have an impaired insulin release due to the absence of a functional clock in pancreatic beta-cells [59]. However, in our case the delayed SREBP1 activation cannot be attributed to a reduced insulin release, since we detect normal glucose and insulin levels in *Bmal1^−/−^* mice at 3 month of age upon RF. Conversely, the expression of REV-ERBα, that is directly regulated by BMAL1, is constantly downregulated. This event leads to the derepression of the Insulin Induced 2 (*Insig2*) gene, encoding a trans-membrane protein that sequesters SREBP proteins to the endoplasmic reticulum membranes, thus interfering with the proteolytic activation of SREBPs, in agreement to what was shown in REV-ERBα−/− mice [16]. SREBP1 activity in the nucleus reflects also the rate of its proteosomal degradation after DNA binding [60], [61], a process that is strongly sensitive to the insulin-mediated inactivation of the glycogen synthase kinase 3 (Gsk3) [62]. In *Bmal1^−/−^* mice the phase of SREBP1 recruitment to DNA is shifted, but we do not observe a longer SREBP1 accumulation on its targets, suggesting that the absence of a functional clock is not significantly altering its degradation process. In conclusion, our results show that besides the nutrient-driven regulation of SREBP1 nuclear accumulation, a second layer of modulation of SREBP1 transcriptional activity exists and is strongly dependent from the circadian core clock. This system allows to fine tune the expression timing of SREBP1 target genes, thus helping to temporally separate the different physiological processes in which these genes are involved. Thus, SREBP1 is situated at the interface of the circadian and the metabolic regulation and its study promises to shed light on the emerging association between diabetes, obesity, sleep, and circadian timing.

## Materials and Methods

### Animals

All animal experiments and procedures were approved by the Swiss Veterinary Office (authorisation VD-1453.4). C57BL/6 male were purchased from Charles River. *Bmal1^−/−^* mice were a kind gift from Dr. Frédéric Gachon and were generated as previously described [63], [64]. 12–14 week old (at time of sacrifice), mice were housed in a 12 h light/12 h dark (LD) regimen for 2 weeks with food and water freely available during night and day. They were then phase-entrained to a 12 hr/12 hr LD regimen with food access between ZT12 and ZT24 for 7 days (ZT = Zeitgeber time; ZT0 is defined as the time when the lights are turned on and ZT12 as the time when lights are turned off). At each ZT2, ZT06, ZT10, ZT14, ZT18 and ZT22 three to five mice were anesthetized with isoflurane and decapitated. Mice were killed under dim red light at ZTs during the dark phase. The livers were perfused with 2 ml of PBS through the spleen and immediately collected. A small piece of liver tissue was snap-frozen in liquid nitrogen. The remaining liver tissue was immediately homogenized in PBS containing 1% formaldehyde for chromatin preparation.

### Chromatin immunoprecipitation (ChIP)

Perfused livers were processed for chromatin preparation as previously described [65]. The chromatin samples from the mice of the same ZT were then pooled, frozen in liquid nitrogen and stored at −80°C. The following antibodies were used: anti-RPB2 (Santa Cruz Biotechnology, H-201), anti-SREBP1 (Santa Cruz Biotechnology, H-160), anti-HNF4 (Santa Cruz Biotechnology, C-19). Chromatin was subjected to immunoprecipitation of Pol II as described [24]. For SREBP1, the samples were diluted ten times in “sonication buffer” containing 50 mM HEPES (pH 7.9), 140 mM NaCl, 1 mM EDTA, 1% Triton X-100, 0.1% Na-deoxycholate, 0.1% SDS and proteinase inhibitors (Roche). 1 ml of diluted chromatin was immunoprecipitated with 10 µg of antibody as described [66]. Briefly, the immune complexes were collected by adsorption to ten µl of protein-A-Sepharose (25% slurry in sonication buffer), pre-blocked with 10 µg/ml of salmon sperm DNA and BSA at 4°C overnight. The beads were washed twice with “sonication buffer”, twice with sonication buffer containing 500 mM NaCl, twice with 20 mM Tris, pH 8.0, 1 mM EDTA, 250 mM LiCl, 0.5% NP-40, 0.5% Na-deoxycholate and twice with TE buffer. The immunocomplexes were eluted with 50 mM Tris, pH 8.0, 1 mM EDTA and 1% SDS at 65°C for 10 min., adjusted to 200 mM NaCl and incubated at 65°C overnight to reverse the cross-links. After successive treatments with 10 µg/ml Rnase A and 20 µg/ml proteinase-K, the samples were extracted with NucleoSpin Kit (Macherey- Nagel). The DNA concentration was determined by fluorometry on the Qubit system (Invitrogen). 10–12 ng DNA were used for the preparation of the library. Libraries for ultra-high throughput sequencing were prepared with the ChIP-Seq DNA sample kit (Illumina) as recommended by the manufacturer.

### RNA isolation and analysis

About 100 mg of snap-frozen liver tissue were used for RNA preparation with the TRIzol reagent (Invitrogen) followed by purification with miRNeasy Mini Kit (Qiagen), according to manufacturer's instructions. For microarray analysis 500 ng of total RNA from each liver sample at the same time point were pooled and analyzed on Mouse Gene 1.0ST arrays according to the manufacturer's instructions (Affymetrix). All statistical analyses were performed with the statistical language R and various Bioconductor packages (http://www.Bioconductor.org). Normalized expression signals were calculated from Affymetrix CEL files using RMA normalization method. For quantitative RT-PCR analysis, the retrotranscription has been done using iScript cDNA synthesis kit (Bio-Rad, Laboratories, Hercules, CA) and following the manufacturer's instructions. The primers sequences are shown in Tables S7 and S8. Real-time monitoring of PCR amplification of cDNA was performed using the FastStart Universal SYBR Green Master (Roche Applied Science, Indianapolis, IN) in an ABI Prism 7900 Sequence Detection System (Life Technologies, Carlsbad, CA). The PCR arbitrary units of each gene were defined as the mRNA levels normalized to the *36b4* and the *Rps9* expression level in each sample using the qBase Software.

### Western blotting

Nuclear extracts were prepared by the NUN procedure as described previously [67], and Western blotting was performed according to standard protocols using the antibody for SREBP1 indicated above. U2AF and Lamin A were used as loading control (anti-U2AF and anti-Lamin A were from Sigma-Aldrich).

### Blood biochemistry

At sacrifice, blood was taken for determination of biochemical parameters and circulating hormones. Insulin levels were determined with ELISA kit from Mercodia, Uppsala, Sweden, following manufacturer's instructions.

### ChIP-seq data analysis and read mapping

At each time point, DNA sequenced reads were mapped to the mouse genome (Mus musculus NCBI m37 genome assembly (mm9; July 2007)) using Bowtie [68] with three mismatches and at most five hits allowed on the genome. When computing genomic read densities, each alignment contributed 1/(total number of hits) to the local density. If several reads in the same library mapped at the same genomic position and on the same strand (redundant tags), we kept only one read for the rest of the analysis. The total numbers of reads per time point are given in Table S1.

### Strand shifting

The mapped reads were shifted to account for the length of the inserts based on the average fragment size, namely 190 184 204 199 202 208 and 176, for each of the seven libraries. The fragment size was divided by two and half of the read length was subtracted resulting in a shift of 55, 52, 62, 60, 61, 64, and 48 nucleotides from ZT02 to ZT26.

### Peak identification and refinement

We fragmented the genome into 500 nucleotide blocks and collected the counts within each block for SREBP1 as well as for input experiments. We kept all blocks for which we had a signal equivalent to 40 tags in at least one time point of the SREBP1 experiment. We log transformed the data after adding 1 pseudo count and quantile normalized both SREBP1 and input experiments. We selected blocks with a log2 signal SREBP1/input greater than 2, i.e. at least a four-fold enrichment of the SREBP1 signal in comparison to the input signal in at least one time point. We repeated this procedure by shifting the block definition of half the length of the blocks and merged the overlapping blocks that passed these criteria.

To define proper “peaks” in these wide regions, we look for shorter regions accounting for most of the counts. For this, we repeatedly consider the two borders along 50 nucleotides, and discard the one with less read counts if we keep 75% of the total reads in the remaining region. This operation is repeated with shorter borders until no further refinement is possible.

### Motif search

We used the MEME suite [21] to identify enriched motifs in the sequences corresponding to the refined peaks. We first performed several motif discovery, on all peaks, and on the subset associated or not with Pol II. We searched for 15 motifs between 6 and 10 nucleotides long. The discovered motifs have been associated to known transcription factors (from the TRANSFAC database) with STAMP. To retrieve these motifs in the subsets where they have not been discovered, we searched for them in all sequences (using FIMO). To assess the relevance of the number of observed motifs in our dataset, we counted the occurrence of the same motifs in random sequences. These sequences are selected in the proximity of the TSS of genes expressed in our samples (among the top 10% in the microarray data). Their size and distance to TSS are in the same range as that of our SREBP1 peaks.

### Cosine fits

Before fitting a cosine function to estimate the amplitude and the phase of the oscillation in a 24 hour period, counts in the refined peaks were quantified and normalized according to the total number of non redundant mapped reads for each given library.

We used the function x(t) = b0+b1*cos(b3+2π*t/24) to perform a least squared fitting of temporal profiles. The parameter b0 represents the mean signal, b1 the amplitude of the oscillation, and b3*24/2π the phase. These parameters were estimated by nonlinear least-squares using the Gauss-Newton algorithm.

For the microarray temporal profile analysis we used the function 


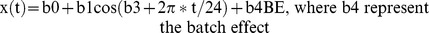


while when we compared WT and *Bmal1^−/−^* mice we used the function
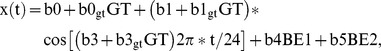
where b4 and b5 represent the different batch effects, GT is a dummy variable that indicates the mouse genotype (WT or KO) and b0_gt_, b1_gt_, b3_gt_ the associated coefficients.

### Data availability

Illumina sequencing data for the ChIP-seq are available at GEO as the GSE48375. Additional processed data and visualization tools are provided at http://cyclix.vital-it.ch.

## Supporting Information

Figure S1 Validation of SREBP1 antibody for ChIP experiments. (A) C57BL/6 mice were fasted 24 hours and re-fed for 12 hours before liver collection for chromatin preparation. SREBP1 binding was tested on two positive control loci, the *Fatty acid synthase* (*Fasn*) and *Low density lipoprotein receptor* (*Ldlr*) promoters. *Neg1* and *Neg2* were used as negative control loci, and correspond to a site specifically recognized by Pol III and to a region between a exon 6 and intron 6–7 of the *glyceraldehyde 3-phosphate dehydrogenase (Gapdh)* gene, respectively. Fold enrichments relative to the negative controls are greater than 40-fold for *Fasn* and about 10-fold for *Ldlr*. (B) In samples processed in absence of antibody none of the tested sequences was significantly enriched. Primer sequences are listed in Table S8.
(PDF)

Figure S2 Temporal relationship of SREBP1 and Pol II profiles and mRNA accumulation of SREBP1 putative target genes. Phase histograms for SREBP1 target genes belonging to clusters A1, A2 and A3. While SREBP1 binding phases are sharply concentrated between ZT14 and ZT18 for all genes, the temporal distribution of Pol II recruitment and mRNA expression is different in the three clusters. The phases are plotted only for genes with an amplitude P-value<0.05 for mRNA.
(PDF)

Figure S3 Metabolic parameters in *Bmal1^−/−^* mice. Body weight (A) and daily food intake (B) were measured in *Bmal1−/−* and control mice at 14 week of age. (C) *Bmal1^−/−^* (red line) and control mice (black line) were fed only during the night for one week before the sacrifice. Plasma glucose levels were measured at the indicated time points (n = 3–6).
(PDF)

Figure S4 Phases distribution of SREBP1 target genes in *Bmal1* ^−/−^ mice. The graph shows the smoothing of phase distributions of the genes belonging to the three clusters (green line for A1, blue line for A2, magenta line for A3). Only genes showing a P-value<0.05 in wild-type mice are plotted.
(PDF)

Table S1 Sequencing data for the ChIP-seq of SREBP1: Number of sequenced and non-redundant tags at each time point.
(PDF)

Table S2 Enrichment of motifs for SREBP1, SP1, NFY and HNF4 in SREBP1 peaks. To estimate the empirical P-Value for motifs discovered by MEME, we randomly selected 1000 groups of 236 regions and counted the number of matches of the indicated motifs in these regions. The random regions were selected to bear the same characteristics as the cluster A regions: their size and distance to TSS are similar to the ones of cluster A regions, and they are close to the TSS of genes expressed in our dataset (expression level above the median expression level of all the transcripts). For all 1000 random groups, we found less matches than in the regions associated to SREBP, showing that these motifs are enriched with an empirical P-Value<0.001.
(PDF)

Table S3 Functional annotation clustering of putative SREBP1 targets using DAVID tools. The annotation links the sites belonging to cluster A to the closest gene irrespective of the distance. In total, 219 out of 236 sites have functional annotation. The genes associated to each functional term that was significantly enriched are shown. P-value = Modified Fisher's Exact Test, B = Benjamini corrected P-value.
(TXT)

Table S4 Functional annotation clustering of putative SREBP1 targets displaying a different combination of binding sites for SP1, NFY and/or HNF4. Functional annotation clustering for the four sub-groups of putative SREBP1 target genes presenting a different combination of binding sites for NFY, SP1 and/or HNF4. The genes associated to each functional term that was significantly enriched are shown. P-value = Modified Fisher's Exact Test, B = Benjamini corrected P-value.
(XLSX)

Table S5 Gene expression profiles in liver tissue of wild type and Bmal1^−/−^ mice for genes reported in Figure 3A and Figure 5A. Microarray gene expression analyses were performed in liver samples from wild type (CCNRC samples) and Bmal1^−/−^ mice (NRC samples). Mice were fed only during the night (ZT12–ZT24) for one week before collecting liver. Reported data are from 2 pools/condition (n = 3–5).
(TXT)

Table S6 Frequency of HNF4 motifs in putative SREBP1 targets with a different temporal expression profile. The percentages refer to the frequency of the HNF4 motif that was discovered by MEME in the regions under SREBP1 peaks of the three cluster of genes with different temporal expression profile. HNF4 motifs were significantly enriched in the peaks of genes expressed between ZT4.4 and ZT13 (P-value<0.02 according to Fisher's exact test).
(PDF)

Table S7 Primer sequences used in qPCR analysis of gene expression.
(PDF)

Table S8 Primer sequences used in ChIP-qPCR experiments.
(PDF)
