# Supplementary figures and images for: Genome-Wide Analysis of SREBP1 Activity around the Clock Reveals Its Combined Dependency on Nutrient and Circadian Signals
Source: PLoS Genet. 2014 Mar 6;10(3):e1004155. doi: 10.1371/journal.pgen.1004155 (PMC3945117; doi:10.1371/journal.pgen.1004155)

**Supplementary Figure S1. Validation of SREBP1 antibody for ChIP experiments.**

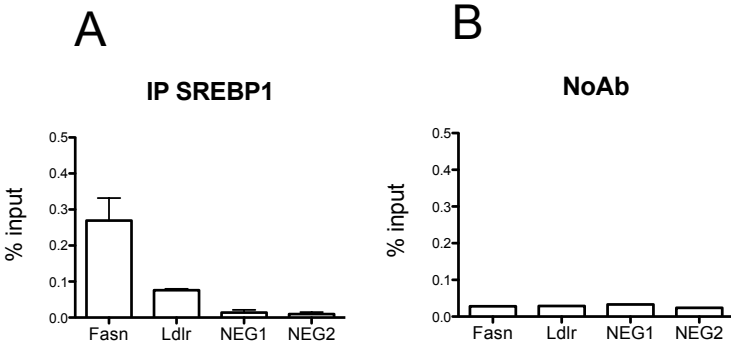

Supplement: Figure S1 — Validation of SREBP1 antibody for ChIP experiments. (A) C57BL/6 mice were fasted 24 hours and re-fed for 12 hours before liver collection for chromatin preparation. SREBP1 binding was tested on two positive control loci, the Fatty acid synthase (Fasn) and Low density lipoprotein receptor (Ldlr) promoters. Neg1 and Neg2 were used as negative control loci, and correspond to a site specifically recognized by Pol III and to a region between a exon 6 and intron 6–7 of the glyceraldehyde 3-phosphate dehydrogenase (Gapdh) gene, respectively. Fold enrichments relative to the negative controls are greater than 40-fold for Fasn and about 10-fold for Ldlr. (B) In samples processed in absence of antibody none of the tested sequences was significantly enriched. Primer sequences are listed in Table S8. (PDF) [file pgen.1004155.s001.pdf]

## Supplementary Figure S3: metabolic parameter in *Bmal1*<sup>-/-</sup> mice

**A**

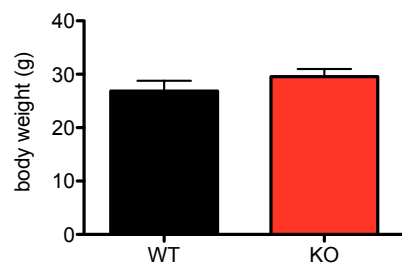

**B**

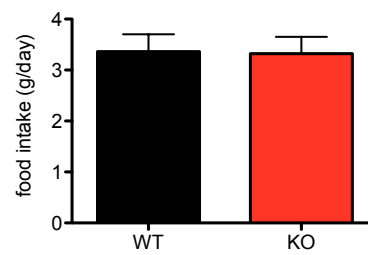

**C**

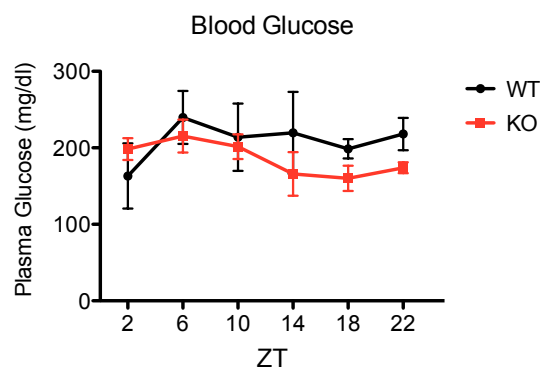

Supplement: Figure S3 — Metabolic parameters in Bmal1−/− mice. Body weight (A) and daily food intake (B) were measured in Bmal1−/− and control mice at 14 week of age. (C) Bmal1−/− (red line) and control mice (black line) were fed only during the night for one week before the sacrifice. Plasma glucose levels were measured at the indicated time points (n = 3–6). (PDF) [file pgen.1004155.s003.pdf]

**Supplementary Figure S4. Phase distribution of SREBP1 target genes in *Bmal1*<sup>-/-</sup> mice**

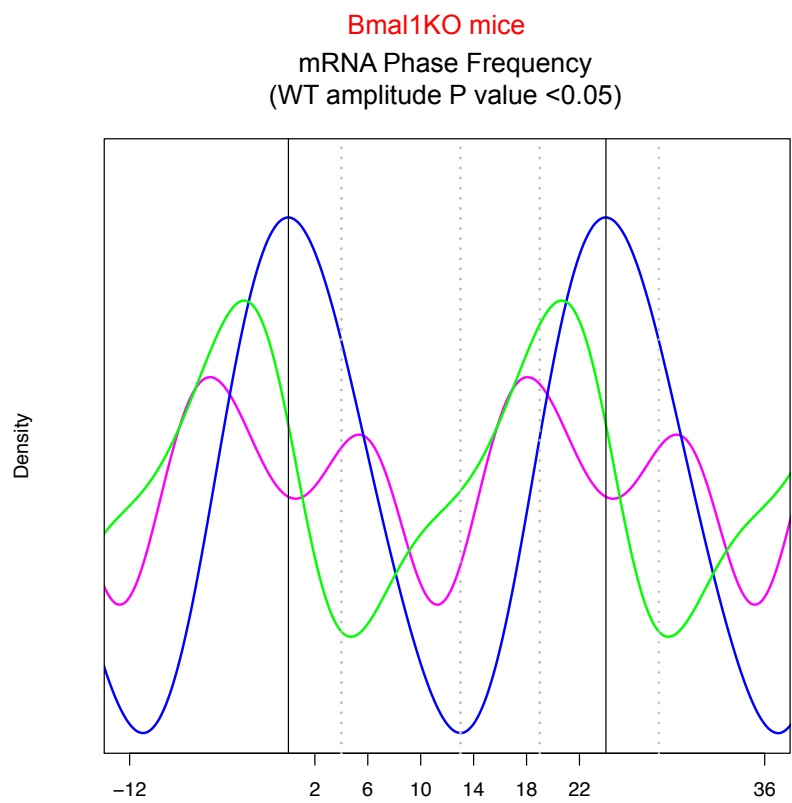

Supplement: Figure S4 — Phases distribution of SREBP1 target genes in Bmal1 −/− mice. The graph shows the smoothing of phase distributions of the genes belonging to the three clusters (green line for A1, blue line for A2, magenta line for A3). Only genes showing a P-value<0.05 in wild-type mice are plotted. (PDF) [file pgen.1004155.s004.pdf]
