# Supplementary material for: Genome-Wide Analysis of SREBP1 Activity around the Clock Reveals Its Combined Dependency on Nutrient and Circadian Signals
Source: PLoS Genet. 2014 Mar 6;10(3):e1004155. doi: 10.1371/journal.pgen.1004155 (PMC3945117; doi:10.1371/journal.pgen.1004155)

Supplementary Figure S2. Temporal relationship of SREBP1and Pol II profiles and mRNA accumulation of SREBP1 putative target genes.

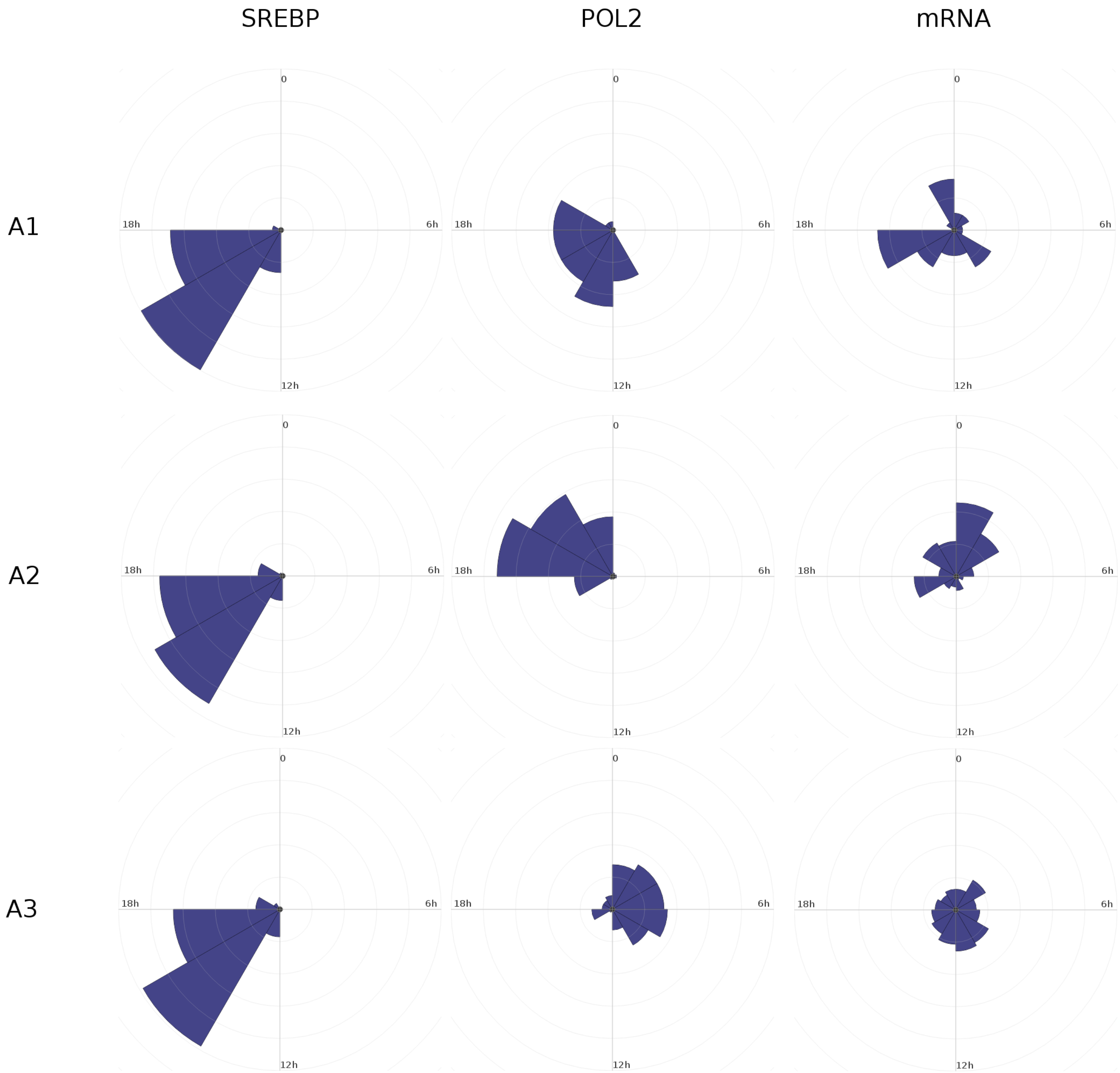

Supplement: Figure S2 — Temporal relationship of SREBP1 and Pol II profiles and mRNA accumulation of SREBP1 putative target genes. Phase histograms for SREBP1 target genes belonging to clusters A1, A2 and A3. While SREBP1 binding phases are sharply concentrated between ZT14 and ZT18 for all genes, the temporal distribution of Pol II recruitment and mRNA expression is different in the three clusters. The phases are plotted only for genes with an amplitude P-value<0.05 for mRNA. (PDF) [file pgen.1004155.s002.pdf]
