# Supplementary material for: Genome-Wide Analysis of SREBP1 Activity around the Clock Reveals Its Combined Dependency on Nutrient and Circadian Signals
Source: PLoS Genet. 2014 Mar 6;10(3):e1004155. doi: 10.1371/journal.pgen.1004155 (PMC3945117; doi:10.1371/journal.pgen.1004155)
Supplement: Table S1 — Sequencing data for the ChIP-seq of SREBP1: Number of sequenced and non-redundant tags at each time point. (PDF) [file pgen.1004155.s005.pdf]

Supplementary Table S1. Sequencing data for the ChIP-seq of SREBP1: number of sequenced and non-redundant tags at each time point

atLeastOneAlign	failed2Align	suppressedDue2m5	reportedAlign	usedReads
4073534 (10.91%)	3032468 (8.12%)	32916444	23781575	22217240
4243058 (10.31%)	3273893 (7.96%)	36612196	27304944	25504542
4742813 (11.49%)	3389202 (8.21%)	36150820	27744795	25937659
4336651 (11.70%)	3042858 (8.21%)	32334405	26474388	24739003
4250645 (11.08%)	3039164 (7.92%)	33758169	29346073	27474051
4245893 (11.61%)	2892161 (7.91%)	32015750	24664390	23058942
