# Supplementary material for: Genome-Wide Analysis of SREBP1 Activity around the Clock Reveals Its Combined Dependency on Nutrient and Circadian Signals
Source: PLoS Genet. 2014 Mar 6;10(3):e1004155. doi: 10.1371/journal.pgen.1004155 (PMC3945117; doi:10.1371/journal.pgen.1004155)
Supplement: Table S2 — Enrichment of motifs for SREBP1, SP1, NFY and HNF4 in SREBP1 peaks. To estimate the empirical P-Value for motifs discovered by MEME, we randomly selected 1000 groups of 236 regions and counted the number of matches of the indicated motifs in these regions. The random regions were selected to bear the same characteristics as the cluster A regions: their size and distance to TSS are similar to the ones of cluster A regions, and they are close to the TSS of genes expressed in our dataset (expression level above the median expression level of all the transcripts). For all 1000 random groups, we found less matches than in the regions associated to SREBP, showing that these motifs are enriched with an empirical P-Value<0.001. (PDF) [file pgen.1004155.s006.pdf]

Supplementary Table S2. Enrichment of motifs for SREBP1, SP1, NFY and HNF4 in SREBP1 peaks.

Motif	Match number in cluster A	Median Random	Max Random
SREBP1	143	15	27
SP1	119	62	85
NFY	72	35	52
HNF4	61	27	47
