# Supplementary material for: Genome-Wide Analysis of SREBP1 Activity around the Clock Reveals Its Combined Dependency on Nutrient and Circadian Signals
Source: PLoS Genet. 2014 Mar 6;10(3):e1004155. doi: 10.1371/journal.pgen.1004155 (PMC3945117; doi:10.1371/journal.pgen.1004155)
Supplement: Table S6 — Frequency of HNF4 motifs in putative SREBP1 targets with a different temporal expression profile. The percentages refer to the frequency of the HNF4 motif that was discovered by MEME in the regions under SREBP1 peaks of the three cluster of genes with different temporal expression profile. HNF4 motifs were significantly enriched in the peaks of genes expressed between ZT4.4 and ZT13 (P-value<0.02 according to Fisher's exact test). (PDF) [file pgen.1004155.s010.pdf]

Supplementary Table S6. Frequency of HNF4 motifs in putative SREBP1 targets with a different temporal expression profile.

PHASE	% HNF4
ZT13-ZT19	14.3
ZT19-ZT28.4	20.9
ZT4.4-ZT13	37.2
