# Supplementary material for: Genome-Wide Analysis of SREBP1 Activity around the Clock Reveals Its Combined Dependency on Nutrient and Circadian Signals
Source: PLoS Genet. 2014 Mar 6;10(3):e1004155. doi: 10.1371/journal.pgen.1004155 (PMC3945117; doi:10.1371/journal.pgen.1004155)
Supplement: Table S7 — Primer sequences used in qPCR analysis of gene expression. (PDF) [file pgen.1004155.s011.pdf]

Supplementary Table S7. Primer sequences used in qPCR analysis of gene expression.

Primer Name	F primer (5'- 3')	R primer (5'- 3')
Aacs	CCAGCCTCATCCTGGAGAC	GGATGTCCCGGTACAGGTC
Clcn6	CTTCACCATTTACCGTCTCCC	AATTCATTTGTCAGGTTGTGCC
Clock1	AGCAGCCAGCTCAGGCC	TGGAGCAACCTAGATGTCTGTAAGA
Cry1	CTGGCGTGGAAGTCATCGT	CTGTCCGCCATTGAGTTCTATG
Cry2	GGTTCCTACTGCAATCTCTGG	GTCATATTCAAAGGTCAAACGGG
Dbp	AAGAACCGGCCAGCTGTCT	GGCTGAGGGCAGAGTTGC
Insig1	GATTACCATCGCCTTCCTAGC	CGTCCTATGTTTCCCACTGTG
Klf10	TCTAGTGTCTCAGTGCTCCC	TTAGTTCCATTTTCCCCTCCG
Obfc2a	GTGCCAAATTTCAGTGAGCC	CCAGTCTGATCACCATTTCCC
Rev-erbα	GACCTTTCTCAGCACGACC	CATCACTGTCTGGTCCTTCAC
Slc25a25	AAGGCTACATCCCCAACATG	AGATAGTACCACAGGCCAGG
Srebp1c	ATGGATTGCACATTTGAAGACATGCT	CCTGTGTCCCCTGTCTCAC
Rps9	GACCAGGAGCTAAAGTTGATTGGA	TCTTGGCCAGGGTAAACTTGA
36B4	AGATGCAGCAGATCCGCAT	GTTCTTGCCCATCAGCACC
