# Supplementary material for: Genome-Wide Analysis of SREBP1 Activity around the Clock Reveals Its Combined Dependency on Nutrient and Circadian Signals
Source: PLoS Genet. 2014 Mar 6;10(3):e1004155. doi: 10.1371/journal.pgen.1004155 (PMC3945117; doi:10.1371/journal.pgen.1004155)
Supplement: Table S8 — Primer sequences used in ChIP-qPCR experiments. (PDF) [file pgen.1004155.s012.pdf]

Table S8. Primer sequences used in ChIP-qPCR experiments.

Primer name	F primer (5'- 3')	R primer (5'- 3')
Aacs SRE	CGTGCCTATGCTCAGAATCC	ACCAGCCCCAAGGAACTTCT
Anks4b SRE	GTCCAGGATTGCAGGTCTTC	GGTTAATCAGTGACGGGGAGT
Clcn6 SRE	CCCTCTCGAGCTCTGGGT	CAGCCAAGTACTGCGTCATG
Fasn SRE	GCGCAGCCCCGACGCTCATT	CGGCGCTATTTAAACCGCGG
Gnat1 SRE	ACCCACGTCAAAGTCACTCA	GGTTAGTGACTGTCCCTCCC
Insig1 SRE	TCAGCCCAGTCTTGTGACAG	CTGTGTCTCCCAAGTGCAAA
Ldlr SRE	GATTCGCAGCCGAGACAC	CCTCACTCGGATCACAGACC
Obfc2a SRE	CTCAGGGAAGGTACGTGACAG	GTTCGCCAGTTGCTGCTT
Slc25a25 SRE	AGCTCTGGTGGAAAATGCAA	GACACCTGCCCTACCCTAAG
Srebf1 SRE2	GTAGCCAATGGGTGCAAGG	CACGTGACCAAAACCAGAGT
Cyp7a1 TSS-1500	CTCTGGCCTAGTGTCATACTCTACCT	GCCAAGCGACCCTCTCA
Cyp7a1 TSS-150	GCTTATCGACTATTGCAGCTCTCT	CTGGCCTTGAACTAAGTCCATCT
Neg1	CTATGGCTTCCTCGCTCTG	TCACGGAAGGTCTGGAGTAG
Neg2	AGCAACTCCCACTCTTCCAC	CAAACCCCCAGTTGCTCTTA
